# Synthesis and Properties of New “Stimuli” Responsive Nanocomposite Hydrogels Containing Silver Nanoparticles

**DOI:** 10.3390/gels1010117

**Published:** 2015-08-28

**Authors:** G. Roshan Deen, Vivien Chua

**Affiliations:** Soft Materials Laboratory, Natural Sciences and Science Education AG, National Institute of Education, Nanyang Technological University, 1-Nanyang Walk, Singapore 637616, Singapore; E-Mail: vi.chua938@gmail.com

**Keywords:** hydrogels, silver nanoparticles, absorption, nanocomposites, swelling

## Abstract

Hydrogel nanocomposites containing silver nanoparticles of size 15–21 nm were prepared by diffusion and *in-situ* chemical reduction in chemically crosslinked polymers based on *N*-acryloyl-*N*′-ethyl piperazine (AcrNEP) and *N*-isopropylacrylamide (NIPAM). The polymer chains of the hydrogel network offered control and stabilization of silver nanoparticles without the need for additional stabilizers. The presence of silver nanoparticles and their size was quantified by UV-Vis absorption spectroscopy and scanning electron microscopy. The nanocomposite hydrogels were responsive to pH and temperature changes of the external environment. The equilibrium weight swelling ratio of the hydrogel nanocomposite was lower in comparison with the precursor hydrogel. Silver nanoparticles present in the nanocomposite offered additional physical crosslinking which influenced media diffusion and penetration velocity. The release of silver nanoparticles from the hydrogel matrix in response to external pH changes was studied. The rate of release of silver nanoparticles was higher in a solution of pH 2.5 due to maximum swelling caused by ionization of the gel network. No significant release of nanoparticles was observed in a solution of pH 7.

## 1. Introduction

Hydrogels are three-dimensional crosslinked (chemical or physical) polymer networks that can absorb large amounts of water and yet remain insoluble. “Stimuli” responsive hydrogels respond to changes in external stimuli such as pH, temperature, ionic strength, electric field, pressure, magnetic field *etc.* [1,2,3,4,5]. The potential responses to these stimuli are changes in shape, volume, phase, and optical properties. The stimuli-responsive volume change of the gels is a result of many factors such as type of monomers, hydrophilic-hydrophobic balance, crosslink density, osmotic pressure, conformation of chemical groups *etc.* These materials are widely applied in targeted drug delivery systems, nanocomposites, protein purification, sensor technology, and enzyme immobilizations [6,7,8,9,10].

The synthesis of nanoparticles is a major area of research, and many methods such as chemical reduction, micro-wave assisted methods, radiation–chemical reductions *etc.* have been widely reported. The chemical reduction process by the methods of co-precipitation, polymer stabilization, and microemulsion has been reported to yield nanoparticles with narrow size distribution [11,12,13]. In recent years the synthesis of nanoparticles within a crosslinked polymer network (hydrogels) by *in-situ* and *ex-situ* chemical reduction has gained considerable research focus in the development of polymer-metal hybrid materials [14,15,16,17]. Such hybrid materials (hydrogel nanocomposites) containing colloidal nanoparticles have wide applications in catalysis, drug-delivery systems, anti-bacterial systems, chemical sensors *etc.* The hydrogel networks provide easy nucleation and growth of nanoparticles without much aggregation or agglomeration which is undesirable for any application [15,16,17]. The presence of nanoparticles in the hydrogel matrix acts at nano-fillers which improves the mechanical properties of the nanocomposites.

Among metal nanoparticles, silver nanoparticles have attracted much research attention in recent years owing to their excellent properties such as, electrical conductivity, bacterial action, optical properties, and catalysis [18,19,20]. They are considered as a non-toxic and environmentally friendly anti-bacterial material. Controlled formation of silver nanoparticles in microgels based on *N*-isopropylacrylamide, acrylic acid, and hydroxyethyl acrylate for the development of photonic crystals has been reported by Kumacheva [20,21,22]. The synthesis of silver and gold nanoparticles in microgels and hydrogels using various chemical methods has been documented in the literature [22,23,24,25]. Thus the development of non-toxic nanocomposite hydrogels containing silver nanoparticles is an active area of research.

In this article, we report the synthesis and characterization of pH- and temperature-responsive silver nanocomposite hydrogels by a facile *in-situ* method. The chemically crosslinked hydrogel is composed of *N*-acryloyl-*N*′-ethyl piperazine (AcrNEP) a pH responsive component [26] and *N*-isopropylacrylamide (NIPAM) a temperature-responsive component [27,28,29,30]. The nanocomposite hydrogel was synthesized by incorporating silver nanoparticles in the gel matrix by diffusion and *in-situ* chemical reduction process. This *in-situ* method of synthesis facilitates the formation of nanoparticles in hydrogels with a low size polydispersity index. The low mesh size of the hydrogel matrix facilitated the growth of silver nanoparticles, and the polymer chain offered stability against aggregation. This study provides the possibility to control the size and quantity of silver nanoparticles in the hydrogel matrix by varying the amount of cationic monomer (AcrNEP) and crosslinker. This type of nanocomposite material is non-toxic and envisaged to possess excellent optical and anti-microbial properties. The influence of external stimuli such as changes in pH on the release of silver nanoparticles from the nanocomposite hydrogel matrix is also described in this report.

## 2. Results and Discussion

### 2.1. Synthesis of Hydrogel and General Characteristics

The hydrogels were prepared by free-radical solution polymerization at 23 °C using an accelerator viz. Tetramethylethylene diamine (TEMED). The solution became viscous and gelled within 10 min after the addition of free-radical initiator potassium persulfate (KPS) and the accelerator. The gels were transparent in appearance and soft in texture. The gel A7N3 was softer than the gel A1N9. The conversion of monomer (*C*^m^°^n^) was estimated based on the following equation and the results are presented in Table 1.

(1) $$C^{mon}=\frac{W_{d}}{W_{i}}\times 100$$

where *W_i_* and *W_d_* are the weight of monomers before polymerization and dry weight of the gel respectively. 

**Table 1:** Monomer feed compositions, conversion, and physical appearance of gels.

Gel	Monomer Feed (mol %)			*C*^mon^ (%)	Appearance	
	AcrNEP ^a^	NIPAM ^b^	MBA		Before polymerization	After polymerization
A1N9	6.90	91.60	1.50	93.45	Clear solution	Clear gel
A7N3	68.95	29.55	1.50	94.26	Clear solution	Clear gel

^a^ molar mass of *N*-acryloyl-*N*′-ethyl piperazine (AcrNEP) = 168 g·mol^−1^; ^b^ molar mass of *N*-isopropylacrylamide (NIPAM) = 113 g·mol^−1.^ C^mon.^ **=** Conversion of monomer. MBA = *N*,*N*'-methylenebisacrylamide

**Figure 1:**
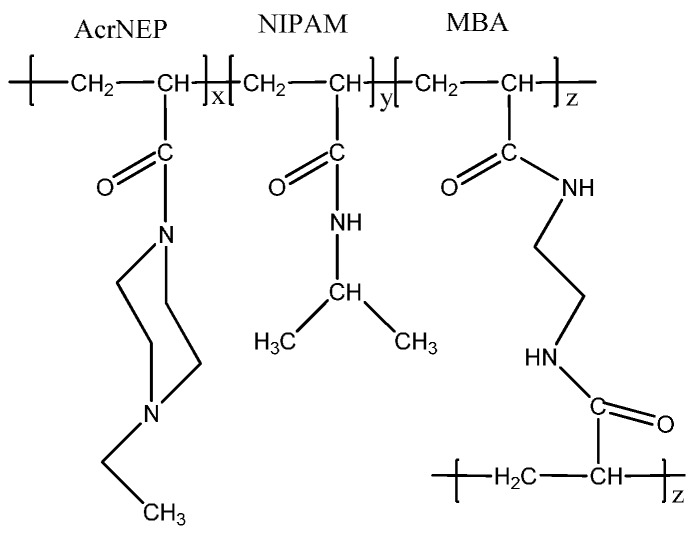
Chemical structure of crosslinked hydrogel.

The conversion of monomers is more than 90% which indicates an efficient crosslinking of the monomers with crosslinker *N*,*N*'-methylenebisacrylamide (MBA). Due to similar reactivity of the acrylic groups of the monomer and crosslinker (CH_2_=CH–CO–) an efficient crosslinking reaction is achieved. Similar observations have been reported for the crosslinking reaction of vinyl caprolactam and hydroxyl ethylmethacrylate with ethylene glycol methacrylate as the crosslinker [31,32]. The chemical structure of the hydrogel is shown in Figure 1.

### 2.2. Fabrication of Silver Nanoparticles by In-Situ Chemical Reduction Method

**Figure 2:**
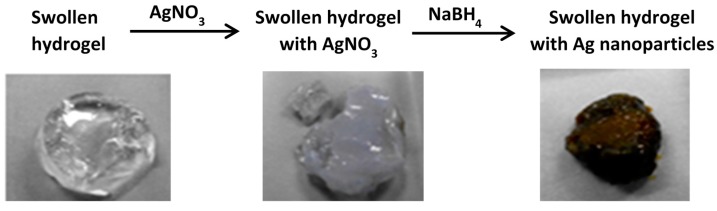
General scheme for the synthesis of silver nanocomposite hydrogels.

The use of hydrogels as templates is an interesting and easy approach in the preparation of nanoparticles/nanocomposites [14,15,16]. Hydrogel silver nanocomposites were prepared by a two-step method at 23 °C. In the first step, the gel was soaked in aqueous AgNO_3_ solution until swelling equilibrium was attained. During this process the silver ions diffused into the gel and formed a 1:1 complex [2,15,16] with the tertiary amine nitrogen of AcrNEP. In the second step, the silver salt loaded gel was chemically reduced by a strong reducing agent, NaBH_4_. During the process of chemical reduction with NaBH_4_ the visual appearance of the gel changed from colorless to yellowish-brown indicating the formation of silver nanoparticles in the gel matrix. The general scheme of the two-step method and the digital image of the gel A7N3 before and after chemical reduction are shown in Figure 2.

The hydrogels reported in this work contain random distribution of AcrNEP and NIPAM repeating units along the copolymer chain with a number of MBA crosslink points. The presence of AcrNEP (ionic monomer) with tertiary amine nitrogen and NIPAM with secondary amine functionality and their random distribution along the copolymer chain acts as nano-templates or nano-reactors for the formation of silver nanoparticles [15,16]. The polymer chains of the hydrogel network offer control and stabilization of the nanoparticles without the need of any further stabilizer.

Further it has been demonstrated that uniform dispersion of metal nanoparticles can easily be synthesized in copolymer hydrogels. The role of AcrNEP in the chemical reduction process cannot be ruled out; however such a reduction process is effective only at higher temperatures [16]. In general, large polymer chains can either act as stabilizing or encapsulating agents for nanoparticle stabilization [33]. The use of natural and synthetic polymers as stabilizers for metal nanoparticles has been reported [34,35].

### 2.3. UV-Vis Absorption Spectra of Hydrogel Nanocomposites

**Figure 3:**
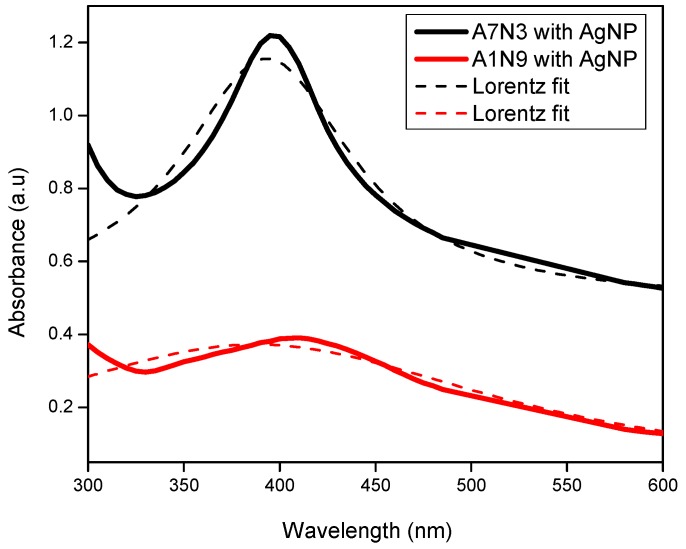
UV-Vis spectra of silver nanoparticles in hydrogel nanocomposites.

The presence of silver nanoparticles in the gels was confirmed by UV-Vis absorption spectroscopy, and the absorption spectra are shown in Figure 3. A distinct absorption maximum is observed at wavelengths 408 nm and 396 nm for the gels A1N9 and A7N3 respectively. This absorption maximum is a characteristic surface Plasmon resonance feature that arises from the quantum size silver nanoparticles present in the gel [36]. Similar absorption peaks have been reported for silver nanoparticles of quantum size [15,16,36,37,38]. The difference in absorption peak position between the gels is attributed to the size polydispersity of silver nanoparticles. In general, very small nanoparticles of uniform size shift the absorption maximum to lower wavelength [36,37,38]. The absorption maximum and the area under the peak exhibited by the gel A7N3 is intense and narrow compared to that of A1N9 which is less intense and significantly broad.

This difference in absorption intensity can be explained as follows: The gel A7N3 contains 68.95 mol % of the ionizable monomer (AcrNEP) units, while A1N9 contains only 6.90 mol %. The presence of higher amount of AcrNEP makes the gel hydrophilic and allows easy diffusion of silver ions and their subsequent reduction by the borohydride ions. The width of absorption peak for gel A1N9 containing silver nanoparticles is comparatively broad which is attributed to the large size polydispersity.

The presence of lower amount of AcrNEP (6.90 mol %) hinders easy diffusion of silver ions and does not offer stabilization to silver nanoparticles against aggregation. The increase in absorption intensity and large peak area therefore represents the formation of a large number of silver nanoparticles. Thus the presence of ionic monomer plays an important role in controlling the size and size distribution of silver nanoparticles in the hydrogel nanocomposites.

Assuming the free particle behavior of electrons the size of silver nanoparticles was calculated using the following formula which is based on modified Mie scattering theory [39],

(2) $$d=\frac{h\;\nu_{f}}{\pi \;\Delta E_{1/2}}$$

where *h* is the Plank’s constant, *v_f_* is the Fermi velocity of electrons in bulk silver (1.39 × 10^6^ m s^−1^), and Δ*E*_1/2_ is the full width at half maxima (FWHM) of the absorption peak. The measured absorbance was fitted with Lorentz distribution fit to obtain the FWHM as shown in Figure 3. The above equation is valid only if the silver nanoparticles are smaller than the mean free path of electrons in the bulk metal. The mean free path of electrons is about 27 nm at room temperature for bulk silver [40]. Using the above formula the average size of silver nanoparticles at 23 °C in A7N3-AgNp nanocomposite was calculated to be ~20 nm.

### 2.4. Surface Morphology of Hydrogel Silver Nanocomposites

**Figure 4:**
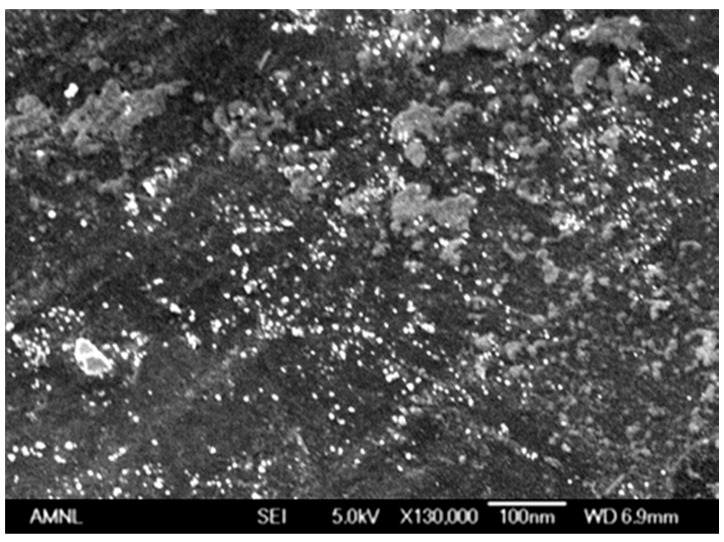
Scanning electron micrograph of gel A7N3-AgNp.

The size and distribution of silver nanoparticles in the gel matrix was studied by scanning electron microscopy, and the micrograph of gel A7N3-AgNP is shown in Figure 4 as a representative example. The presence of finely dispersed silver nanoparticles throughout the gel matrix is clearly seen. The average size of the silver nanoparticles is in the range 15–21 nm as calculated from the micrograph. The porous morphology of the gel network and the presence of charged monomer units along the copolymer chain are believed to be the factors in stabilizing the silver nanoparticles against aggregation. Such domains of nanometer length scale allow the growth of silver nanoparticles. The porosity of gel network and hydrophilicity of polymer chain allow easy diffusion of small molecules (silver nitrate, sodium borohydride, and water) into the network for rapid reduction. The hydrogels facilitate easy diffusion of small ions for complexation and subsequent chemical reduction and these important properties are widely accepted in sensor technology and catalysis [15,16,20].

The immobilization of silver nanoparticles throughout the hydrogel matrix is due to strong location of silver ions in the network. This is possible due to complexation of silver ions with nitrogen and oxygen atoms of the polymer chain. The tertiary amine nitrogen of the piperazine ring of AcrNEP is known to form co-ordinate complexes with both univalent and divalent metal ions [41]. The crosslinker content of the gels was fixed at 1.5 mol % to allow easy diffusion of small ions such as borohydride into the gel for chemical reduction. Gels with high crosslinker content are rigid and restrict diffusion of ions leading to non-uniform distribution of nanoparticles in the gel matrix.

### 2.5. Effect of pH on Swelling of Hydrogel Nanocomposite

The gels prepared in this work were responsive to pH changes of the external solution. The gels swelled considerably more in acidic solutions than in basic solutions. The equilibrium swelling ratio as a function of the pH of the external solution is summarized in Table 2. Maximum equilibrium swelling is observed for the gels in a solution of pH 2.5, which is attributed to complete protonation of the tertiary amine (^+^N–CH_2_–CH_3_) of AcrNEP. The protonation leads to the formation of fixed electrical charges on the polymer network that repel by Coulombic interactions. This leads to an osmotic pressure gradient between the gel matrix and external solution which causes the gel to swell. The pK_a_ of AcrNEP is 4 and maximum swelling is expected in solutions of pH lower than 4.

**Table 2:** Equilibrium weight swelling ratio (WSR^Eq^) of gels as function of solution pH at 23 °C.

Gel	WSR^Eq^					WSR^Eq pH = 2.5^/WSR^Eq^ ^pH = 12^
	pH = 2.5	4.0	6.5	10.0	12.0	
A1N9	7.14	5.81	5.20	4.52	3.54	2.02
A7N3	34.25	13.21	11.63	7.02	6.26	5.47

Cationic gels prepared from vinylimidazole, dimethyl aminoethylmethacrylate, vinyl pyridine *etc.* have been reported to show maximum equilibrium swelling below their respective pK_a_ values [42,43,44]. The visual appearance of the swollen gels in acidic solution was transparent and homogeneous. This indicates a favorable thermodynamic interaction between the polymer chains and water molecules. In solutions of basic pH the tertiary amines are not ionized and the gels do not show significant swelling. Therefore by directly varying the pH of the external solution the swelling capacity of these cationic gels can be easily modulated.

The influence of ionizable monomer viz. AcrNEP on the equilibrium swelling of gels is clearly observed in Table 2. The equilibrium swelling ratio of the gel A7N3 that contains 68.95 mol % of ionizable group is 35 while for the gel A1N9 that contains only 6.90 mol % of ionizable groups it is only 7.1. An increase in the ionizable group increases the protonation sites on the gel network and hence the charge density leading to a large increase in swelling in solution of low pH. The swelling transition defined by WSR^Eq^ ^pH = 2.5^/WSR^Eq^ ^pH = 12^ also increases with increase in ionic group content of the gels which is attributed to the pH dependent protonation and de-protonation in acidic and basic solutions respectively.

### 2.6. Effect of Silver Ions and Silver Nanoparticles on the Swelling of Nanocomposite

The swelling behavior of the gels in water (pH = 6.5) before and after chemical reduction process was also studied by measuring the weight swelling ratio at equilibrium. The swelling experiment was conducted in deionized water to eliminate any possible interference by ions present in buffer solutions on the swelling of gels.

The inorganic ions can reduce the hydrogen bonding sites in the gel by acting as physical crosslinkers. This additional physical crosslinking effect can decrease the swelling capacity of the gel.

**Figure 5:**
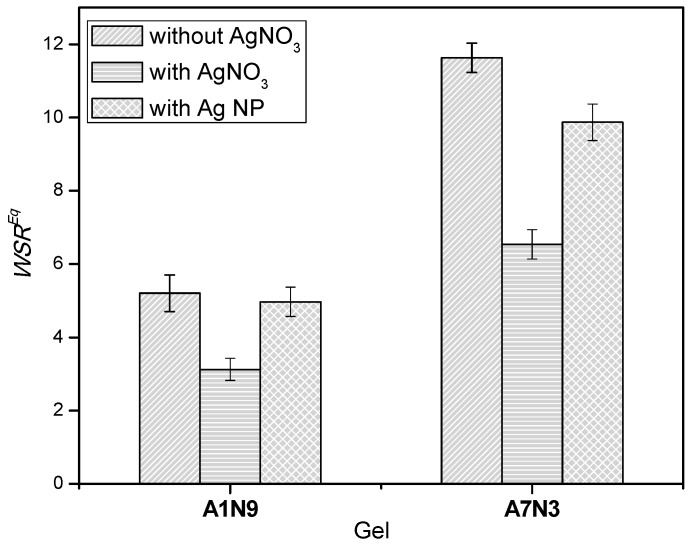
Equilibrium weight swelling ratio of gels and silver nanocomposites in water (pH = 6.5) at 23 °C.

The change in equilibrium weight swelling ratio of the gels before and after chemical reduction is shown in Figure 5. The influence of silver salt and the silver nanoparticles on the bulk properties of the gels is observed, and the order of swelling ratio is as follows: hydrogel > gel with silver nanoparticles > gel with silver salt.

It is evident that upon incorporation of AgNO_3_ within the gel by the diffusion method, the swelling ratio of the gels decreases considerably. The swelling ratio for the gel A7N3 decreases from 11.63 to 6.91, and for the gel A1N9 it decrease from 5.20 to 3.12 when loaded with silver salt. This reduction in swelling ratio for both gels is ~40%. This marked decrease in swelling ratio is attributed to the additional physical crosslinking (ionic crosslinking) of the gel network caused by the silver ions. This behavior is similar to the influence of chemical crosslinker on the swelling behavior of gels *i.e.*, with an increase in the amount of chemical crosslinker the swelling of gels decreases.

Interestingly, the swelling ratio of gels increases after the chemical reduction processes indicating the loss of ionic crosslinking caused by the silver salt. The swelling ratio of the gels containing the silver nanoparticles is lower than that of the gels without the nanoparticles. This again is a consequence of the silver nanoparticles that hinders the diffusion of water into the gel matrix. Similar reduction in swelling of silver nanocomposites hydrogels has been reported earlier [15,16]. Therefore, hydrogel nanocomposites with the desired degree of swelling can be developed by varying the amount of metal nanoparticles in the gel matrix.

### 2.7. Effect of External Temperature on Swelling of Hydrogel Nanocomposite

**Figure 6:**
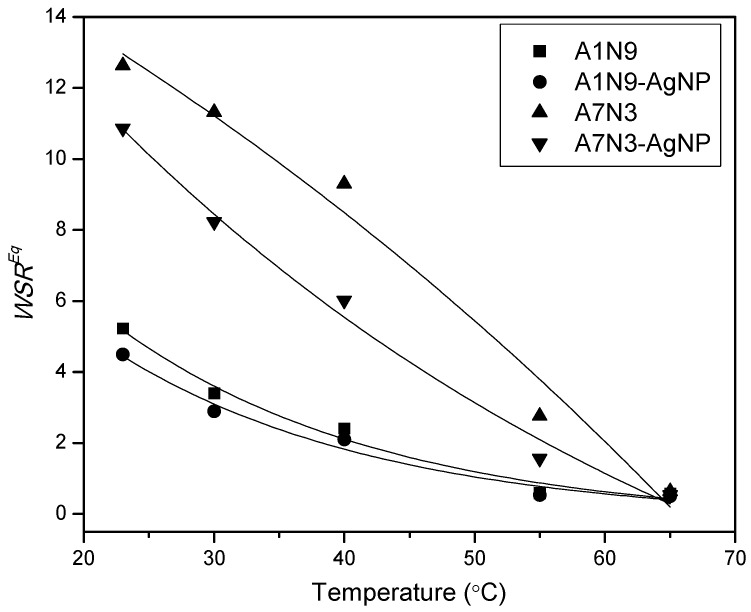
Effect of temperature on the swelling of gels and nanocomposite gels in water. Lines are an eye guide.

The effect of external temperature on the swelling of gels and nanocomposite gels in water (pH = 6.5) was studied and the results are shown in Figure 6.

With increasing temperature from 23 °C to 65 °C, the swelling ratio of the gels and nanocomposite gels drops continuously. This decrease in swelling at high temperature is a signature of the thermo-responsive property of hydrogels. The gels contain two types of polymer components *viz*. PNIPAM which is a temperature sensitive component with lower critical solution temperature (LCST) around 32 °C in water [28], and PAcrNEP which is a pH sensitive component with no LCST. At 23 °C the gels and the nanocomposite gels are in the fully swollen state with a thermodynamically good interaction with the surrounding water molecules. As the temperature is increased from 23 °C to 65 °C, the interaction with water molecules weakens which leads to enhanced polymer-polymer interaction (hydrophobic interactions) in line with Gibbs’ free energy equation as [28].

(3) $$\Delta G=\Delta H^{mix}-T\Delta S$$

where *H*^mix^, *S*, and *T* are the enthalpy of mixing, entropy, and temperature in K.

The volume phase transition temperature (VPT) of the gels A1N9 and A7N3 were measured to be 36 °C and 64.8 °C respectively which were in agreement with the LCST of the corresponding linear polymers [45]. This increase in VPT of A7N3 is due to the presence of a high amount of AcrNEP which is a hydrophilic comonomer. In general, hydrophilic comonomers increase the VPT of gels while hydrophobic comonomers decrease it [27]. Therefore above the VPT, complete shrinking of the gels is expected owing to hydrophobic interactions [27,28]. Interestingly, the nanocomposite gels also exhibit a decrease in swelling ratio with increase in temperature and the trend is more pronounced for A7N3-AgNp than A1N9-AgNp. This is because nanocomposite A7N3-AgNp contains a high amount of silver nanoparticles which act like additional physical crosslinkers that facilitate the collapse of the gel [15,24]. 

### 2.8. Effect of Silver Nanoparticles on the Water Diffusion Mechanism of Nanocomposite

**Figure 7:**
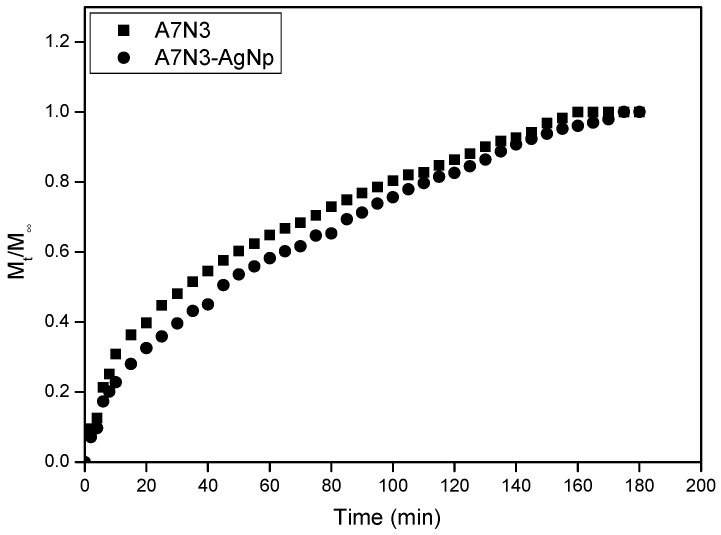
Normalized water sorption isotherm of hydrogel and hydrogel nanocomposite.

The influence of silver nanoparticles on the water sorption kinetics of the gel A7N3 was studied to understand the water transport mechanism. This gel and the corresponding nanocomposite gel were chosen for this study due to their good swelling properties. The normalized water uptake (*W*_t_/*W*_∞_) isotherms measured in water at 23 °C are shown in Figure 7. In general, the gel and the nanocomposite (A7N3-AgNp) show a steep water uptake during the first 20 min followed by a gradual slow increase.

Two different processes describe the diffusion of solvent into the gel *viz*. (i) diffusion of solvent and (ii) advancement of the swollen-deswollen boundary. In order to determine the type of water sorption mechanism of the gel and the nanocomposite gel, the initial swelling data was fitted to the following empirical equation [46] in the range *M_t_*/*M*_∞_ ≤ 0.6:

(4) $$\frac{M_{t}}{M_{\infty }}=k\;t^{n}$$

where *M_t_* and *M*_∞_ are the mass of water measured at time *t* and time infinity (equilibrium swelling) respectively, *k* is a characteristic constant of the gel, and *n* is the diffusion exponent that describes the transport mechanism of penetrate. This generalized expression combines both Fickian and non-Fickian mechanisms for thin polymer slabs or discs.

The values of *n* and *k* were directly calculated from the gradient and intercepts of a log (*M_t_*/*M*_∞_) *versus* log t plots and the results are presented in Table 3. For the gel without the silver nanoparticles the value of n is equal to 0.63. This corresponds to non-Fickian type diffusion of solvent molecules into the gel in which the rate of solvent diffusion and polymer relaxations is comparable. For the nanocomposite hydrogel the value of n is lower and is equal to 0.53. This value almost corresponds to Fickian type of diffusion in which the rate of diffusion of solvent is lower than polymer relaxation [47]. This change in diffusion process for the nanocomposite hydrogel is expected due to the presence of silver nanoparticles. The silver nanoparticles acts as additional physical crosslinks and retard the solvent diffusion. Similar behavior of solvent diffusion has been reported for silver nanocomposite hydrogels based on *N*-isopropylacrylamide and sodium acrylate [15]. 

**Table 3:** Water transport characteristic parameters measured at 23 °C.

Gel	*n*	*k*	*D* (10^−8^/cm^2^·s^−1^)	*v* (10^−3^/cm·g^−1^)
A7N3	0.62	0.17	15.72	2.59
A7N3-Ag Np	0.53	0.10	13.23	1.50

*n*: mode of transport of penetrant, *k*: characteristic constant of the gel, *D*: diffusion coefficient, *v: penetration velocity*.

The role of silver nanoparticles as additional crosslinks in the nanocomposite hydrogel was further confirmed by calculating the diffusion coefficient and penetration velocity and the results are also presented in Table 3. The diffusion coefficient *D* for the short-time scale was evaluated from the slope of *M_t_*/*M*_∞_ ≤ 0.6 *versus* square-root of *t* plot using the following expression [46,48,49]:

(5) $$\frac{M_{t}}{M_{\infty }}=\frac{4}{l}\sqrt{\frac{D\;t}{\pi }}$$

where *l* is the initial length of the dry gel and *t* is the time. 

The penetration velocity [50] of water into the hydrogel and the nanocomposites hydrogel was calculated using the following expression:

(6) $$v=\frac{1}{2\;{\text{ρ}}_{w}A}\frac{M_{t}}{M_{\infty }}$$

where ρ_w_ is the density of water at 23 °C (0.9975 g·cm^−3^), and *A* is the area of one face of the thin hydrogel. The factor 2 accounts for water diffusion through both faces of the disc. 

The diffusion coefficient of water for the nanocomposite hydrogel is lower by 16% compared to the hydrogel without silver nanoparticles. The penetration velocity of water in the nanocomposite hydrogel also shows a marked decrease by 35% compared to hydrogel without silver nanoparticles. The marked decrease in diffusion and penetration velocity of water in the nanocomposite hydrogel is attributed to the presence of silver nanoparticles which hinder water penetration into the gel. It is believed that the silver nanoparticles increase the entropy of the system and hinder the hydrogen bonding sites causing lower expansion of the gel network.

### 2.9. State of Water in Swollen Hydrogel Nanocomposite

The effect of silver nanoparticles on the states of water in fully swollen hydrogel nanocomposite was also studied. In general, the state of water in the hydrogels can be divided into free water, freezing bound water, and non-freezing bound water [51]. Free water exists in hydrogel networks without any hydrogen bonding with polymer chains and has a similar transition temperature, enthalpy, and DSC thermogram similar to that of pure water. Freezing bound or intermediate water exhibits weak interactions with the polymeric network of the gels. The non-freezing water or bound water forms hydrogen bonds with the polymeric network and does not show any endothermic peak in the temperature range −70 °C to 0 °C. 

The precursor hydrogel A7N3 and nanocomposite A7N3-AgNp showed a melting peak in the range 0 °C to 7 °C (DSC thermogram not shown). This indicates the existence of free and freezing bound water in the swollen hydrogel nanocomposite network in addition to bound water. The presence of unbound water which is comprised of free and freezing water was estimated by following the standard equation [52],

(7) $$W_{bound}\text{ (\%)}=EWC-(W_{f}+W_{fb})$$

where *EWC*, *W_f_*, and *W_fb_* are the equilibrium water content, free water, and freezing bound water respectively. 

The amount of free water content in the total water present in the gels was calculated as the ratio of the DSC endothermic peak area of the swollen gel to that of the melting endothermic heat of fusion of pure water (333.3 J·g^−1^) using the following equation. This equation assumes that the heat of fusion of free water in the gel is similar to that of ice.

(8) $$W_{bound}\text{ (\%)}=EWC-\left(\frac{\text{Δ}H_{endo}}{\text{Δ}H_{ice}}\right)$$

where Δ*H_endo_* and Δ*H_ice_* are the heat of fusion of free water in the gels and ice respectively. The results are summarized in Table 4. 

**Table 4:** Water content (free and bound) in gel and nanocomposite gel.

Sample	Unbound Water (%)	Bound Water (%)	Unbound Water/Bound Water
A7N3	82.15	17.85	4.60
A7N3-AgNp	75.60	24.40	3.10

The amount of unbound water in hydrogel nanocomposite is lower than the precursor hydrogel A7N3 that does not contain silver nanoparticles. The presence of silver nanoparticles in the nanocomposite acts as physical crosslinkers which increase the rigidity of the gel network. This increase in rigidity resembles that of hydrogels with a high crosslink density. As a result of this, a decrease in unbound water content of the hydrogel nanocomposite is observed. This is also further highlighted by a decrease in the ratio of unbound water/bound water content of the hydrogel nanocomposite.

### 2.10. Effect of External pH on the Leaching of Silver Nanoparticles from Nanocomposite

**Figure 8:**
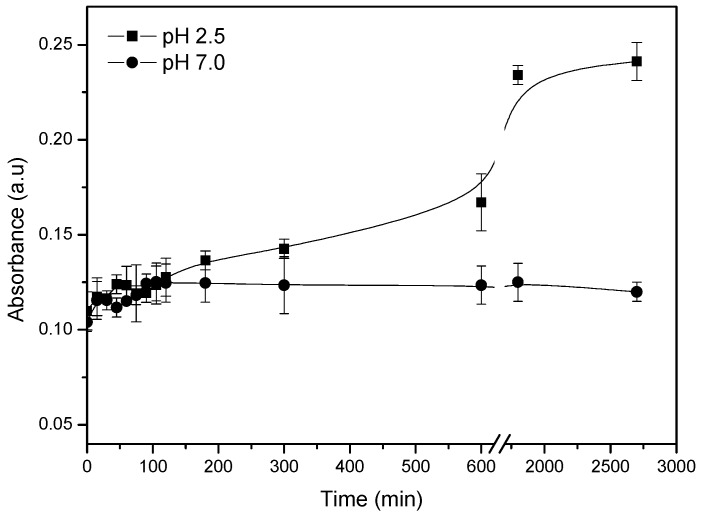
Effect of pH on the release of silver nanoparticles from hydrogel nanocomposite.

The influence of external pH on the leaching of silver nanoparticles from the gel A7N3 was studied by UV-Vis absorption spectroscopy at 23 °C. The gel containing the silver nanoparticles was soaked in solutions of pH 2.5 and pH 7 under very slow stirring (200 rpm) and the absorbance of solution at 406 nm was recorded at intervals of 15 min. The results are shown in Figure 8. The insert in Figure 8 shows the change in absorbance of solution for the first 300 min.

No significant change in absorbance is observed for the gel soaked in a solution of pH 7 even after 24 h. This is attributed to the weak swelling of the gel which does not favor diffusion of silver nanoparticles from the gel to the external solution. However, for the gel soaked in a solution of pH 2.5, a significant and gradual increase in absorbance of the external solution is clearly observed. This increase in absorbance is due to the presence of silver nanoparticles in the external solution as a result of leaching from the gel. In a solution of pH 2.5, the gel is in the fully swollen state due to the fixed charge repulsions of the polymer network.

In this swollen state, the silver nanoparticles in the gel easily diffuse out to the external medium through the many pores in the swollen gel. This process of diffusion is illustrated in Figure 6. The ionic monomer content in the gel and the nature of the swelling medium thus play a vital role in the leaching of nanoparticles from the gel. This type of leaching could be prevented by decreasing the mesh size of the gel by increasing the amount of chemical crosslinker.

## 3. Conclusions

A dual-responsive hydrogel nanocomposite containing finely dispersed silver nanoparticles was successfully prepared by a facile two-step approach. The silver nanoparticles were stabilized by the polymer network. The nanocomposite hydrogel was responsive to changes in external pH and temperature. The presence of silver nanoparticles acts as additional physical crosslinkers and influenced the media diffusion and penetration velocity. By this study, it has been demonstrated that stimuli-responsive nanocomposite hydrogel systems of the desired swelling capacity can be fabricated by a facile *in-situ* chemical reduction process, and stability of the nanoparticles can be achieved by fine-tuning the hydrogel network. The developed hydrogel nanocomposites are non-toxic and could find applications as anti-bacterial surfaces. 

## 4. Experimental Section

### 4.1. Materials

*N*-isopropylacrylamide (NIPAM) (Aldrich, Saint Louis, USA) was purified by recrystallization in toluene/hexane (80:20 *v*/*v*) mixture. *N*,*N*-methylenebisacrylamide (MBA) (Aldrich), *N*-ethylpiperazine (Aldrich), triethylamine (Aldrich), potassium persulfate (KPS) (Aldrich), tetramethylethylenediamine (TEMED) (Aldrich), sodium borohydride (NaBH_4_) (Aldrich), and silver nitrate (AgNO_3_) (Aldrich) were used as received. Tetrahydrofuran (THF) (Merck, Darmstadt, Germany) was purified by distillation and stored over molecule sieves of pore size 3 Å (Aldrich). Deionized water was obtained from Barnstead purification system and was used for all aqueous sample preparations. The pH of the deionized water was 6.5. The buffer solutions of pH 2.5, 4.0, and 6.5 were prepared by adjusting 0.1 M sodium acetate solution with acetic acid to the desired pH. The buffer solutions of pH 10.0 and 12.0 were similarly prepared using sodium hydrogen phosphate and disodium hydrogen phosphate. The ionic strength of all the buffer solutions was fixed at 0.1 M.

### 4.2. Synthesis of Acryloyl Chloride and N-Acryloyl-N′-ethylpiperazine (AcrNEP)

Acryloyl chloride and AcrNEP were synthesized according to the procedure described in the literature [2,26].

### 4.3. Synthesis of Crosslinked Copolymer Hydrogels

Copolymer hydrogels of AcrNEP and NIPAM with two different monomer feed were synthesized by free radical solution polymerization at 23 °C. The crosslinker content in the gels was fixed at 1.5 mol %, and the total monomer concentration was kept at 11 wt %. The synthesis of gel A1N9 is described as follows: AcrNEP (6.90 mol %), NIPAM (91.60 mol %), and MBA (1.5 mol %) were dissolved in deionized water (8 g) in a clean test tube. The content was degassed by bubbling dry nitrogen gas for 10 min to expel any dissolved oxygen. KPS (0.01 g/L in 1 mL water), and TEMED (0.01 g/L in 1 mL water) were added to the monomer solution and the test tube was sealed using Para film. Polymerization was conducted at 23 °C. The solution turned viscous and finally to a clear gel in about 10 min and the reaction was allowed to continue for 24 h. The transparent soft solid gel was cut into small discs (thickness = 0.3 cm and diameter 0.7 cm) and washed in water for 1 day to remove any unreacted monomers. The gel discs were dried in a vacuum oven at 40 °C for 2 days until constant weight was attained. The gel A7N3 was similarly prepared. The monomer feed compositions and the physical appearances of gels are summarized in Table 1.

### 4.4. Fabrication of Silver Nanoparticles in the Hydrogels

Silver nanoparticles were fabricated in the hydrogel by the following *in-situ* method: The dry hydrogel disc (for example A7N3, thickness = 0.3 cm, diameter = 0.7 cm) was soaked in 30 mL of aqueous AgNO_3_ solution (5 × 10^−3^ M) until equilibrium swelling was achieved (usually 1 day). The silver nitrate loaded gel was then soaked in 30 mL of aqueous solution of NaBH_4_ (10 × 10^−3^ M) for 30 min. During this *in-situ* chemical reduction process, the silver salt in the hydrogel was reduced to colloidal silver particles. The transparent gel turned yellowish brown in color during this reduction process. The gel was repeatedly washed in water and dried in a vacuum oven at 40 °C until constant weight was recorded. 

### 4.5. Characterization of Hydrogels

Equilibrium swelling of the gels and nanocomposites was studied gravimetrically to determine their response to pH and temperature changes. The samples were soaked and equilibrated in buffer solutions of various pH (2.5, 4.0, 6.5, 10.0, and 12.0) at 23 °C until swelling equilibrium was reached (~1 day). The swollen weight was recorded after carefully removing the excess surface water by using a damp Kim-wipe towel. The equilibrium weight swelling ratio *WSR^Eq^* was calculated using the following equation,

(9) $$WSR^{Eq}=\frac{W_{s}-\;W_{d}}{W_{d}}$$

where *W_s_* and *W_d_* are the equilibrium swollen weight and dry weight of the sample respectively. 

For water uptake and diffusion study, the samples were cut into thin rectangular slabs (area ~1.5 cm^2^, thickness ~0.3 cm) and placed in glass vials containing 5 mL of water (pH = 6.5). The vials were placed in a thermostatic water bath at 23 °C, and the weight of samples was recorded at different time intervals (*W_t_*). All measurements were repeated three times and the average value was recorded.

The UV-Vis absorption spectra of the gels containing silver nanoparticles were recorded on a Cary-1E UV-Vis spectrophotometer. Samples for this measurement were prepared as follows: About 5 mg of the dry hydrogel disc was dispersed in 5 mL of water at room temperature. The dispersion (~3 mL) was transferred into a clean quartz cell (Helma, path-length = 1 cm), and the absorbance was recorded in the wavelength range 300–700 nm. 

The morphology of freeze-fractured gels was characterized using a Jeol JSM 6700 Field Emission Scanning Electron Microscope (FESEM). The freeze-fractured and dried hydrogel samples were coated with a thin layer of gold in vacuum for 10–20 s for the FESEM measurements. Samples containing the silver nanoparticles were not coated with gold. 

The amount of free and bound water content in the fully swollen gels was determined using a Perkin-Elmer Diamond Differential Scanning Calorimeter (DSC). The swollen gel samples were heated from −20 °C to 20 °C with a ramp rate of 2 °C under a nitrogen atmosphere. 
